# Surface Structured Polymer Blend Fibers and Their Application in Fiber Reinforced Composite

**DOI:** 10.3390/ma13194279

**Published:** 2020-09-25

**Authors:** Dan Pan, Siqi Liu, Licheng Wang, Junfen Sun, Long Chen, Baozhong Sun

**Affiliations:** 1 State Key Laboratory for Modification of Chemical Fibers and Polymer Materials, College of Materials Science and Engineering, Donghua University, Shanghai 201620, China; dan.pan@dhu.edu.cn (D.P.); 2180476@mail.dhu.edu.cn (S.L.); 1199041@mail.dhu.edu.cn (L.W.); junfensun@dhu.edu.cn (J.S.); 2 College of Textiles, Donghua University, Shanghai 201620, China

**Keywords:** surface structured fiber, polypropylene fiber, fiber-reinforced material, melt spinning

## Abstract

Melt-spun surface structured fiber could be a large-scale versatile platform for materials with advanced surface function and local properties. Fibers with distinct surface and bulk structures are developed by tailoring the viscosity ratio and blend ratio of polymer component using the melt-spinning method. Spherical bulge and fibril groove structured fibers are obtained in different viscosity ratio and blend ratio systems. The interfacial bonding between fiber and matrix is improved due to the mechanical interlocking between the structured surface and matrix. The low-viscosity second phase stays as a spherical droplet even in high content. The second phase in matched- and high-viscosity ratio cases is deformed into fibril like droplet which causes an in-situ fibration of the second phase in polymer blend fiber with an enhanced mechanical property. This method provides a simple route to developing polymer materials with surface structure and appropriate mechanical properties to apply in textile and polymer fiber-reinforced composite materials.

## 1. Introduction

The booming infrastructure activities along “The Belt and Road” line, especially in the areas with an extreme environment like saline-alkali soil, tjaele, island, and high daily temperature variation, require more application of advanced polymer fiber-reinforced composites to overcome the effect of the harsh natural environment on the duration of infrastructure materials. Polypropylene (PP) fiber has become an attractive option for application in extreme conditions due to its chemically inert, appropriate thermal properties, low weight, high specific strength, hydrophobia, and low cost [1,2,3]. However, disadvantages including weak interfacial adhesion between fibers and matrix, smooth surface, and low modulus of elasticity all need to be improved to meet the application of fibers in extreme conditions. Thus, lots of works have focused on using oxidative, plasma polymerization, gamma radiation, polymer grafting methods to modify the fiber surface, aiming to increase the surface roughness, surface area, and interaction between fibers and matrix [4,5,6,7,8,9,10,11]. These methods still need to solve environmental and process problems.

Recently, newly developed polypropylene/polyethylene hybrid fibers have achieved an enhancement of mechanical properties up to 40% in the fiber-reinforced cementitious composites [12,13]. This polymeric hybrid fiber possesses rugosity surface and self-fibrillation, which promote the mechanical interactions between fiber and the cement. This study inspired us that this is a great method to build surface structure during fiber spinning rather than post-chemical or physical treatment. However, the formation mechanism and tailoring method of surface morphology are not mentioned in references [12,13]. Melt-spun fibers with gradient morphologies and unique surface patterns were reported in our previous work [14,15,16]. Bump structures are observed on the fiber surface due to the rheological behaviors of dispersed phases under the parabolic flow field during melt-spinning, and (sub) micron grooves are observed on the fiber surface after dissolving the second domains. A similar method had been used to fabricate porous structure in PP fibers by selective extraction of Poly (vinyl alcohol) (PVA) from PP/PVA blend fibers [17].

Theoretically, the morphology of polymer blends is controlled by the microrheological behaviors of the second phase, including deformation, break-up, coalescence, and migration under the action of hydrodynamic and interfacial tension during processing flow [18,19,20]. The microrheological behaviors of the second phase during capillary flow and fiber spinning have been investigated by many researchers based on theoretical and experimental studies [16,21,22,23]. The viscosity ratio and blend ratio have been approved to be effective parameters for tuning the morphologies and properties of polymer blends [24,25,26]. The melt processing can generate morphologies ranging from dispersed drops to fibers to lamella to co-continues structures in the bend ratio of 0–50% [20]. Tsebrenko and co-authors’ studies also showed that specific fiber formation could be controlled by tailoring the rheological and interfacial properties [23,27,28,29]. Our previous studies found that unique gradient morphologies with long thin fibrils near the surface region and short large droplets near the center region of the filament, as well as the inverse pattern, can be produced by tunning the viscosity ratio of polymer blends and shear flow in capillary [14,15]. The formation mechanisms of gradient microstructures were also investigated based on capillary theory. These studies give us a possible approach to precisely tailor the surface structure of polymer blend fibers based on the gradient structure.

The purpose of this paper is trying to tailor the surface topology of fiber during melt spinning based on the formation mechanism of gradient structure and improving the interfacial adhesion between fiber and matrix. The effect of viscosity ratio and blend ratio of polymer blend on the surface morphologies of fibers are investigated. The bonding strength between fiber and matrix is evaluated by a single fiber pull-out test. The effect of the morphology of the dispersed phase on the tensile strength of the produced fiber is also studied.

## 2. Experiments

### 2.1. Materials

Polypropylene (HP560P) with melt flow index of 13 g 10 min^−1^ (230 °C/2.16 kg) was purchased from Taiwan Plastics & Polymers. Three types of polystyrene (PS) with different melt flow indices were selected to satisfy the variation of viscosity ratio of PS to PP. PS1:PS with melt flow of 45 g 10 min^−1^ was synthesized according to the method listed in Table S1 of Supporting Information. PS2:PS (PG-33) with melt flow of 8 g 10 min^−1^ was obtained from CHIMEI Taiwan. PS3:PS (Polystyrol^®^ 144 C) with melt flow of 3.5 g 10 min^−1^ was obtained from BASF Corporation. Epoxy resin (Spurr, SPI-CHEM) with vinyl cyclohexene dioxide (ERL-4221), diglycidyl ether of polypropylene glycol (DER 736), nonenyl succinic anhydride (NSA), and dimethylamino ethanol (DMAE) were purchased from Structure Probe, Inc. West Chester, PA, USA.

### 2.2. Sample Preparation

PP and PS granules were mixed on a twin-screw extruder (D = 35, L/D = 48) at 230 °C. The PS/PP polymer mixtures were then melt-spun on a lab-scale spinning line with a single screw extruder (D = 20, L/D = 25) at a spinning temperature of 250 °C. The as-spun fiber was quenched in a water bath installed 90 cm beneath the spinneret and with a taken-up at speed of 40 m/min. A cross air blow with a velocity of 7 m min^−1^ was applied at room temperature. The morphology of the PS/PP blend fiber was controlled by changing the weight fraction of the PS and viscosity ratio. The investigated weight fraction of PS was set at 2%, 4%, 8%, and 20%.

### 2.3. Rheological Measurements

The rheological properties of the polymers were measured using a rotary rheometer (MARS 60, Thermo Fisher Scientific HAAKE. Waltham, MA, USA) with a 20 mm diameter parallel plate fixture. The shear viscosity of each polymer was measured at 250 °C. After loading the samples into the cell, the temperature was maintained at 250 °C for 3 min to melt the polymers and subsequently equilibrated for 30 s prior to the rheological measurements. Steady sweeps were performed with shear rates ranging from 10^−2^ to 10 s^−1^. The time sweeps were conducted at a shear rate of 1 s^−1^, see Supplementary Materials Figure S1. All rheological measurements were repeated three times to demonstrate reproducibility. PP and PS were dried at 80 °C overnight under vacuum before any use.

### 2.4. Mechanical Properties Analysis

**Figure 1:**
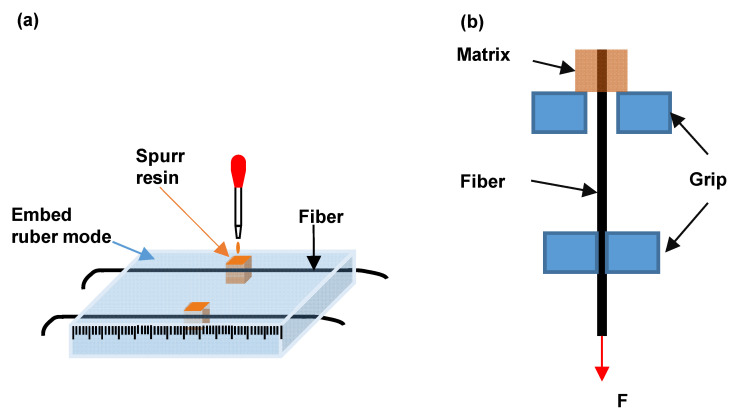
Schematic diagram of the single fiber pull-out test (**a**) fiber embedding; (**b**) fiber pull-out.

In order to evaluate the effect of the surface structure of fiber on the interfacial strength of fiber/resin composite, a single fiber pull-out test was performed on a single fiber strength testing machine (Shanghai Xinxian Instrument Co., Ltd., Shanghai, China) based on reference as shown in Figure 1 [30]. PS/PP blend fibers were carefully fixed in a rubber mode and embedded about 2 mm in epoxy resin. The epoxy resin was solidified at 60 °C for 24 h. The free end of the fiber was held in a standard fiber test grip. The embedded side was restrained using two edges of grip. The embedded length and fiber diameter were measured for each sample under an optical microscope. The tensile stress of the single fiber was also tested at the same mechanical testing machine to investigate the relationship between the bulk structures and mechanical properties of fiber.

The interfacial shear strength *τ* is defined as [30]:

(1) $$\tau =\frac{F}{2\pi rl},$$

where F is the failure force, r is the radius of fiber, and l is the embedded length.

### 2.5. Morphology Characterization

The surface structure morphologies of fibers were analyzed with a scanning electron microscopy (SEM, FEI Quanta 250, Thermo Fisher Scientific. Waltham, MA, USA). The bulk morphologies of fibers were obtained from optical microscopy (Leica, DM2700, Wetzlar, Germany). In the case of bulk structure morphology, the fiber was carefully sliced into small pieces with a blade to obtain the longitudinal sections of the blend fibers under optical microscopy. At least five samples from different parts of prepared fibers were selected to make sure of the structural reproducibility.

## 3. Results and Discussion

### 3.1. Surface Morphology of Fiber

**Figure 2:**
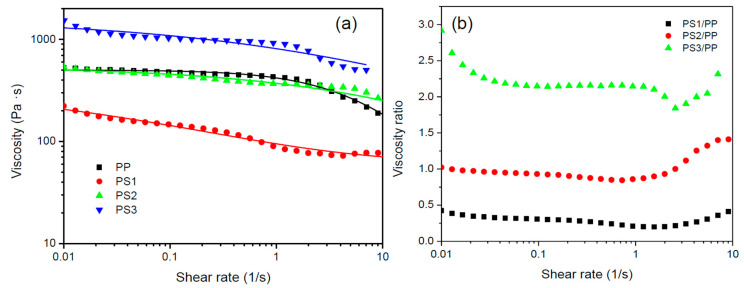
The shear viscosities of polymers (**a**) and viscosity ratio of polymer blends; (**b**) as a function of shear rate.

The viscosity ratio is one important factor that influences the morphology of polymer blends [14,31]. The deformation and break-up of dispersed droplets can be evaluated by capillary number Ca, which describes the capacity for deformation or break-up of droplets under the mechanical equilibrium between viscous forces and interfacial tension during flow [32,33]. The critical capillary number Ca_C_ (calculated from ${\mathrm{logCa}}_{c}=-0.506-0.0994\;\mathrm{logp}+0.124\log^{2}\left(p\right)-\frac{0.155}{\log-0.611}$) is the function of viscosity ratio p for the continuous stretching and breaking up of the dispersed phase during shear flow [34]. Dispersed phases with different viscosities in one polymer matrix have different abilities to break-up, deform, and migrate during flowing. The effect of viscosity ratio and other parameters on the microrheological behaviors, such as droplet deformation, break-up, and immigration, and on the final morphology of polymer blend fibers has been studied in our previous studies [14,15,35] and many other experimental studies [26,36,37,38,39], where the viscosity ratio shows a dominant effect on the morphology of polymer blends. In this article, three sets of polymer blends with three different viscosity ratios were designed to build a surface structure on fiber. Figure 2 shows the viscosities and the viscosity ratio of the polymers versus shear rate at 250 °C. Both PP and PS show typical shear thinning behavior. The zero shear viscosity (η_0_) of the polymers was obtained through curve fitting using the three-parameter Bird-Carreau model [40]:

(2) $$\eta =\frac{\eta_{0}}{{(1+{\left(\lambda \dot{\gamma }\right)}^{2})}^{\frac{1-n}{2}}}$$

where η is the shear viscosity, $\dot{\gamma }$ the shear rate, n the power-law index, and λ the relaxation time. The η_0_ of PP, low-viscosity PS (PS1), medium-viscosity PS (PS2), and high-viscosity PS (PS3) were estimated to be ~502, ~366, ~545, and ~1578 Pa·s, respectively. The viscosity ratios of PS1/PP, PS2/PP, and PS3/PP were 0.2~0.5, 0.8~1.5, and 2~3, respectively. The other fitting parameters of Equation (2) are given in Table S2 of the Supporting Information. The dilatant behavior of polymer blends was not observed, as shown in Figure S2. In the process, the die swell was also negligible due to the dominance of take-up action in agreement with the previous experiments and models [41,42]. 

**Figure 3:**
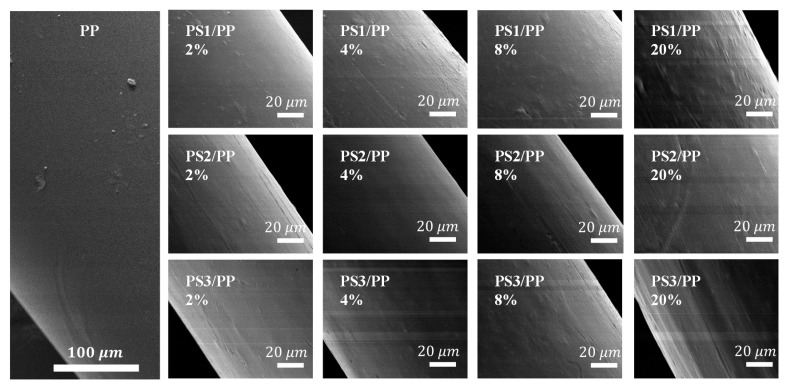
Surface micrographs of PS/PP fibers produced with different viscosity ratios and blend ratios.

The surface morphologies of fibers melt-spun from PS/PP blend with different viscosity ratios and PS contents are shown in Figure 3. It can be seen that the surface of neat PP fiber is smooth. The PS/PP blend fibers show a bump structure on their surface. In the case of low-viscosity ratio PS1/PP blend fibers, the bump structure is shown as spherical bulge morphology. More bulge structures were observed with the increase of PS1 content. In the case of middle-viscosity ratio PS2/PP blend fiber, the surface is relatively smooth, but the ellipsoidal and fibril bulges are still visible on the surface. Fibril-like bulges were observed in the high-viscosity ratio system PS3/PP. The bump microstructures on the surface become clearer in PS/PP blends with a high content of PS phases. It is clearly illustrated that a change in the viscosity ratio for the same blend composition has a significant effect on the surface morphology of polymer blends. The low-viscosity ratio PS1/PP blend fibers show a relatively rough surface compared to PS2/PP and PS3/PP blend fibers. The emerged microstructure on the PS1/PP blend fiber surface mainly comes from the increasing mobility of the PS phase, which leads to the immigration of the PS phase to the fiber surface region, thus resulting in an enrichment of PS phases on the fiber surface. This phenomenon was also reported in other polymer blends during the melt extrusion process [14,15,43]. It must be mentioned that, in the case of the PS1/PP blend with 20% low-viscosity PS1 phases, the spinnability is poor and cannot take-up easily. The samples studied in this case were taken up by hand. This indicates that low-viscosity PS phases in the blend are not good for the stable formation of fiber due to the fast phase separation, especially in the blend with a high content of the second phase, and so the weight fraction of the second phase should keep a low value to support the stable spinning process.

**Figure 4:**
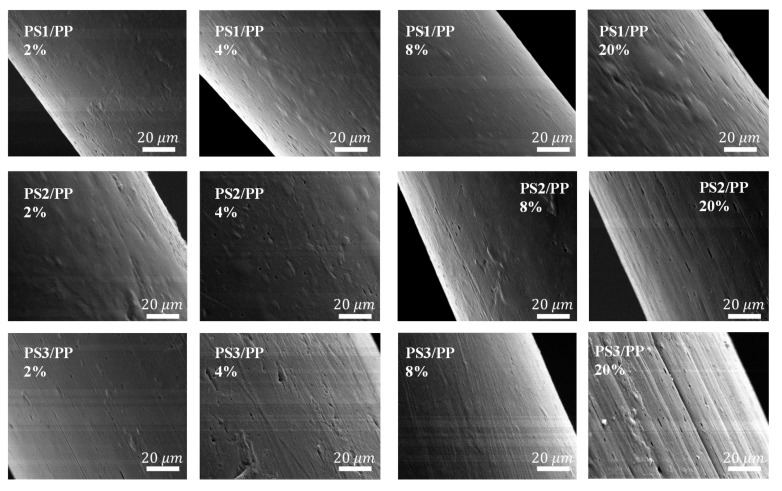
Surface micrographs of PS/PP fibers produced with different viscosity ratios and blend ratios after dissolving the surface PS domains.

**Figure 5:**
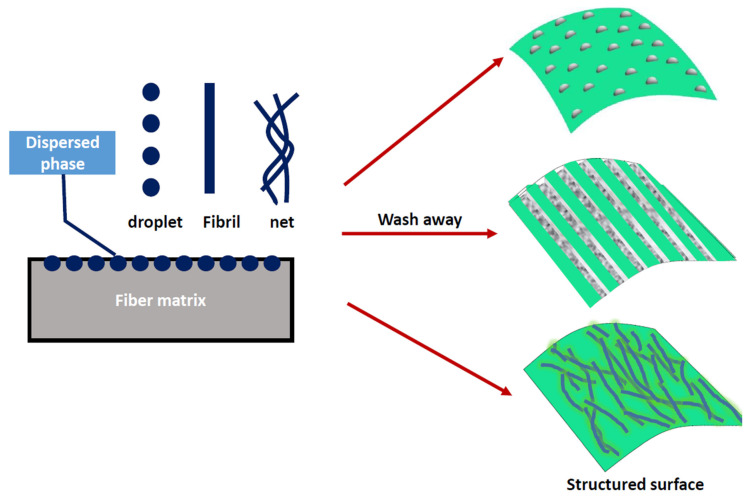
Schematic graph for using dispersed phases as a model to build structures on fiber surface.

In order to further study the microstructure and the surface morphologies of fibers, PS/PP blend fibers were etched with tetrahydrofuran to dissolve the PS phases on the fiber surface and the corresponding SEM morphologies are given in Figure 4. Black holes and fibril grooves were observed after extracting the PS domains on the fiber surface. Short black holes were mostly found in the case of the PS1/PP blend fiber and low addition PS in the high-viscosity ratio system. Long fibril grooves were mostly found in the case of the PS2/PP and PS3/PP blend fibers with a high content of PS. The fibril grooves on the PS3/PP blend fibers with 20 wt% PS3 were larger and longer than those on PS2/PP blend fibers. The bump structures became clearer after the etching process. Thus, those PS/PP blend fibers with low PS contents with a smooth surface can also possess a rough surface after dissolving the PS phases. It can be also found that some spherical-like and fibril-like bulge structures were still left on the fiber surface, which indicates some dispersed PS phases were wrapped by the PP matrix. The embedded PS phases under the fiber skin pushed the matrix outside to form into shaped bulges. Therefore, the PS phase can act as a model to build microstructure by controlling the shape of PS domains; see the schematic graph in Figure 5. The spherical and fibril-like bulge structures, grooves [15], and bundle-like microfiber structure [17] could be generated on the fiber surface. Usually, these kind of surface-structured fibers are produced by wet-spun [44], electrospun [45], how drawing [46] processes. The melt-spun method provides an effective way to modify the surface structure of polymer materials. The geometry of the revealed surface structures depends on the formed shape of the dispersed phase resulting from the deformation, break-up, and coalescence of dispersed phases under the action of hydrodynamic and interfacial force during melt processing. Reference [47] shows that it is possible to produce evenly-distributed dispersed phases with uniform size when the flow field and performed blends are set uniform. On the other hand, the micro- and nano-structures, such as microsphere, nanorod, and ultrathin fibers, could also be created by dissolving the matrix polymers [29,48,49].

### 3.2. Interfacial Bonding between Fiber and Resin

**Figure 6:**
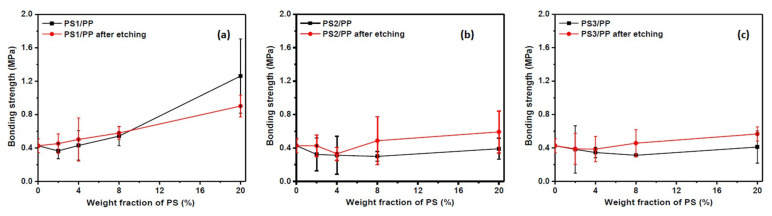
The interfacial bonding strength of different fiber-matrix systems (**a**) PS1/PP; (**b**) PS2/PP; (**c**) PS1/PP.

Studies show that the increase in roughness on the fiber surface facilitates mechanical anchoring between fiber and matrix materials [9,50]. Some microstructures with rough surface shown in Figure 3 and Figure 4 seem to be beneficial to the interfacial bonding between fiber and resin composite. The interfacial mechanical property analyzed from a single fiber pull-out test was evaluated to investigate the effect of surface microstructure on the interfacial bonding between fiber and matrix; see Figure 6. The interfacial bonding strength showed a huge variation in different viscosity ratio systems. In the case of the low-viscosity ratio PS1/PP system, the addition of 2 wt% of PS caused a decrease in the bonding strength between fiber and matrix. The addition of more PS phases increased bonding strength, and the bonding strength reached the maximum value at PS content of 20 wt% with about 196% increased bonding strength compared with neat PP fiber. The PS1/PP fiber after dissolving the surface PS1 phase presented higher bonding strength than that before dissolving treatment, except in the case of PS1/PP with 20 wt% of PS1. In the case of PS2/PP and PS3/PP blend fiber-matrix systems, the addition of PS phases did not increase the bonding strength, and the etching procedure did not give any positive effect on the interfacial bond strengthening at low PS content cases. However, the average bonding strength still had a slight increase in the case of PS2/PP and PS3/PP with 20 wt% of PS content. Basically, the bonding strength increased with increasing PS contents in most cases. It should be mentioned here, the interface of fiber-matrix becomes complicated with the emergence of surface structures. Even small bubbles in the unfilled etched holes and gaps between fiber and matrix will result in a huge variation in the bonding strength. So, one can see big measurement errors in the bonding strengths of different single fiber-matrix composites. However, the average value still tells the trend.

**Figure 7:**
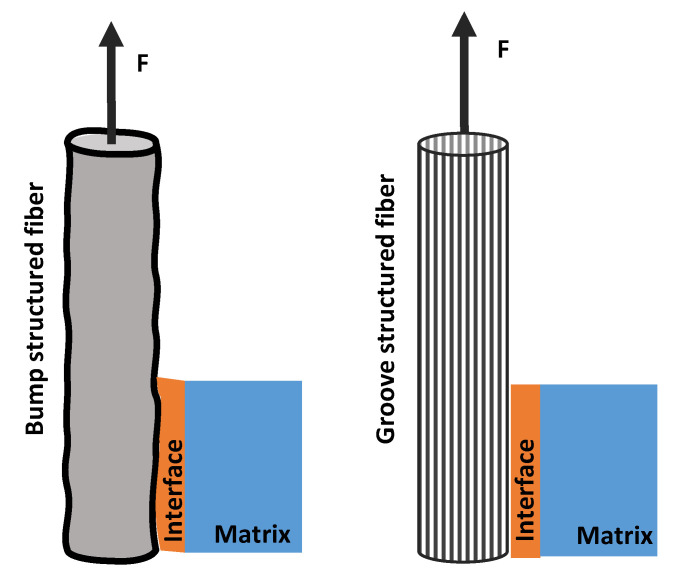
Schematic mechanism diagram of single fiber with different surface microstructure pull out from the matrix.

It was noted that the blend fibers with low PS content cannot create a high roughness surface to improve the interfacial bonding between fiber and resin. Only PS1/PP blend fibers with 8 wt% and 20 wt% of PS which have a large number of bump structures on the surface can improve the interfacial bonding with the matrix. On the other hand, the PS/PP blend fibers after dissolving out the surface PS parts all had a positive effect on the strengthening of interfacial bonding in studied cases. From the results reported so far, the microstructure on the fiber surface has a dominant effect on the interfacial bonding between fiber and matrix. The PS1/PP fibers with spherical bulge structures on the fiber surface had a better effect on the strengthening of interfacial bonding than the PS/PP fibers with long groove structure during the single fiber pull-out test. It must be mentioned that the single fiber pull-out test cannot exactly reflect the mechanical interlocking between fiber and matrix, it just reflects the mechanical interlocking in the direction of pull-out force which is along the fiber axis; see Figure 7. However, the breaking force could act on randomly dispersed fibers in all directions during the crack of a composite. The groove structure on the fiber surface cannot be bonded well with the matrix and even a slip could happen in the direction of the pull-out force. However, the fiber with a bump structure could grip the matrix in any direction.

### 3.3. Bulk Structures and Mechanical Properties of Polymer-Blend Fibers

**Figure 8:**
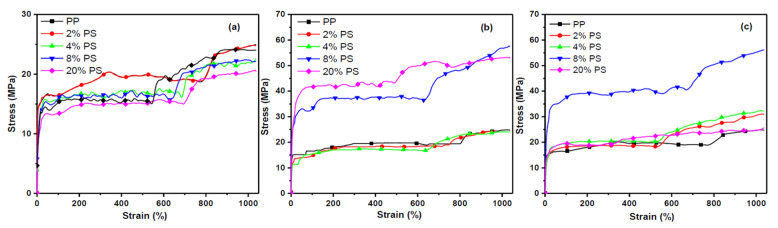
The stress-strain curve of PP and PS/PP blend fibers, (**a**) PS1/PP; (**b**) PS2/PP; (**c**) PS3/PP.

**Figure 9:**
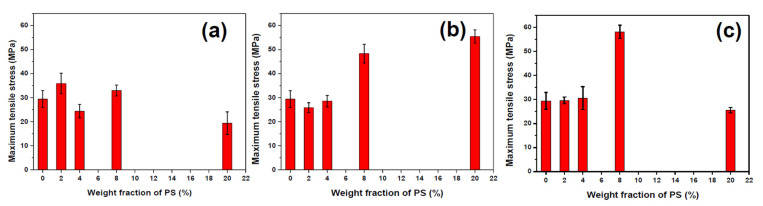
The maximum tensile stress of PS/PP blend fibers: (**a**) PS1/PP; (**b**) PS2/PP; (**c**) PS3/PP.

**Figure 10:**
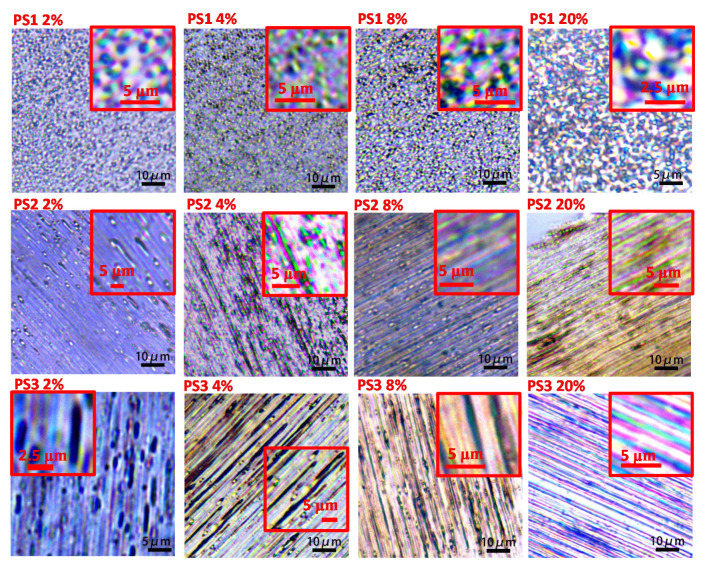
The optical micrographs of PS/PP blend fibers.

Usually, the addition of the second phase would raise a big challenge in the mechanical properties of materials. To satisfy the application of surface structured fiber in the industrial field, it is necessary to optimize the bulk structure of the blend system for minimization of the negative effect of the second phase on the tensile properties of fiber. The effect of the addition of different viscosities and fractions of PS phases on the tensile properties were simply evaluated, and the stress-strain curve and maximum tensile stress of fibers were plotted in Figure 8 and Figure 9. Figure 10 shows the longitudinal sections of fibers which give a full view of matrix-dispersed microstructure morphologies to support a clear understanding of the structure and property relationship in blend fibers. In the PS1/PP blend fiber with low-viscosity PS1, the maximum tensile stress of PS1/PP fibers was close to or slightly lower than that of pure PP fiber, which shows a negative effect on the mechanical properties of fibers. The PS1 phases in the matrix were shown as spherical droplets of about 1–2 μm in diameter, see Figure 10. In the PS2/PP blend fiber with matched viscosity, the maximum tensile stress of PS/PP blend fiber with 2% and 4% of PS was lower than pure PP fiber. The PS2 phases were shown as deformed droplets and short fibrils in low PS addition. However, the PS2/PP blend fiber with 8% and 20% of PS, which showed as long fibrils, possessed higher tensile stress than that of pure PP fiber. In the PS3/PP blend fiber with high-viscosity PS3, the tensile stress of PS3/PP blend fiber was all higher than that of pure PP fiber, especially in the case of 8% of the PS phase. However, the tensile stress decreased a lot when the PS content reached 20%. This phenomenon could be related to the higher uniformity of the fibril diameter created in this case, which has been also observed for other researchers [24,29]. The morphology of the PS3 phase in Figure 10 was shown as a fibril shape and became thinner and longer with the increase of PS3 content. From the results reported so far, we can conclude that the droplet-matrix morphology had a negative effect on the mechanical property of polymer blend fiber in our studied cases. However, the uniformly distributed fibril-matrix morphology showed a positive effect on the mechanical property. These phenomena were also found in other polymer-blend fibers [24,51,52].

**Figure 11:**
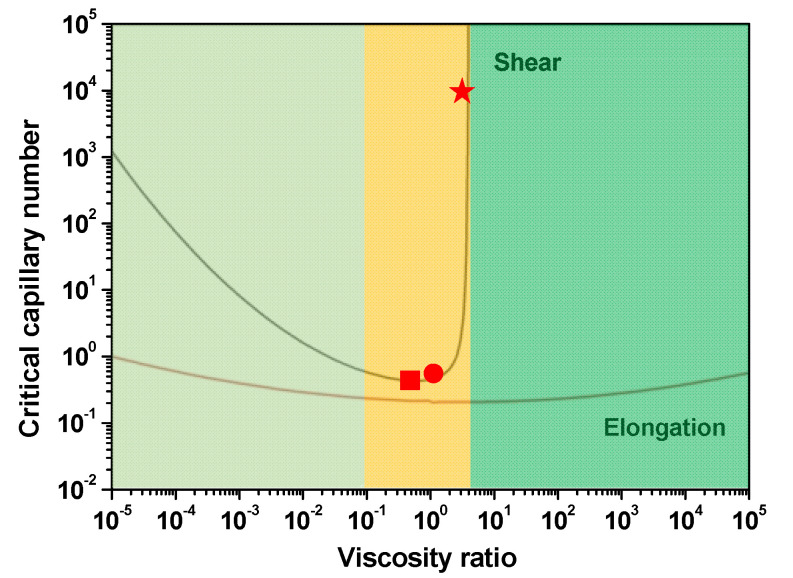
The critical capillary number of dispersed phases as a function of the viscosity ratio of the polymer blend. The red square symbol represents PS1/PP, the red circular symbol represents PS2/PP, the five-star symbol represents PS3/PP.

Figure 10 shows the optical micrographs of PS/PP blend fibers. It can be seen that an in-situ fibration of the PS phase happened in the case of the high-viscosity ratio polymer blend system. The in-situ fibril PS and PP composite was obtained with enhanced tensile property. Figure 11 shows the critical capillary number of dispersed phase at different viscosity ratios, which describes the ability of different viscous phases to deform and break-up in the flow field [34]. The high-viscosity PS3 phases in PP matrix with high critical capillary numbers have a low possibility to break up into small droplets during processing and will continually deform into fibrils. On the other hand, the low-viscosity PS phases have more possibility to break up into droplets. The interesting thing is that even PS1 and PS2 have a small difference in critical capillary numbers, but these two kinds of dispersed phases show a huge difference in deformation behavior. The PS1 phase always stays spherical even in the high loading of the PS phases, and the droplet diameter is independent of the content of the PS phase in the studied loading range. These phenomena deserve further detailed studies, which will be emphasized in the next article. The in-situ fibration of the second phase can also happen in polymer-blends with compatibilizer [53] and polymer-blends after the stretching process [54]. The in-situ fibration of the second phase resulting from tailoring the rheological properties and interfacial properties of polymers have been also studied by many researchers [16,23,24,27,28,29,48,52,55] based on experimental and mathematic studies. These studies suggest that it is possible to build surface structures and improve the mechanical properties of polymer-blend fibers simultaneously.

## 4. Conclusions

Different microstructures were built on polymer-blend fiber surfaces by the variation of viscosity ratio and blend composition during melt-spinning. The low-viscosity PS phase dispersed as spherical droplets in the PP matrix, leading to spherical bulges on PS/PP fiber surface under the action of the hydrodynamic force. The surface of the matched-viscosity ratio PS/PP blend fiber showed relatively smooth morphology. The high-viscosity PS phase was shown as deformed droplets and stretched fibrils, leading to a fibril groove structure on the fiber surface. The bump microstructures on the fiber surface become clear in high PS loading cases and clearer after the dissolving process. The interfacial bonding between fiber and resin matrix was improved with surface structured fiber. The spherical bulge surface structures showed a better effect on the strengthening of interfacial bonding than long fibril groove structures in a single pull-out test. The fibril PS phases have an in-situ strengthening effect on the fiber materials, thus providing an effective way to produce polymer materials with advanced properties.

## Supplementary Materials

The following are available online at https://www.mdpi.com/1996-1944/13/19/4279/s1, Figure S1: The shear viscosities of polymers as a function of time at 250 °C, Figure S2: The shear viscosities of polymer blends as a function of shear rate at 250 °C, Table S1: The reaction recipe of suspension polymerization of PS, Table S2: Fitted parameters of the three-parameter Bird-Carreau model for PS/PP blends.
