# Supplementary material for: Surface Structured Polymer Blend Fibers and Their Application in Fiber Reinforced Composite
Source: Materials (Basel). 2020 Sep 25;13(19):4279. doi: 10.3390/ma13194279 (PMC7579542; doi:10.3390/ma13194279)
Supplement: Supplementary file 1 [file materials-13-04279-s001.pdf]

materials

Supplementary

Surface Structured Polymer Blend Fibers and Their Application in Fiber Reinforced Composite

Dan Pan [1,2], Siqi Liu [1], Licheng Wang [1], Junfen Sun [1], Long Chen [1,*] and Baozhong Sun [2,*]

[1] State Key Laboratory for Modification of Chemical Fibers and Polymer Materials, College of Materials Science and Engineering, Donghua University, Shanghai 201620, China; dan.pan@dhu.edu.cn (D.P.); 2180476@mail.dhu.edu.cn (S.L.); 1199041@mail.dhu.edu.cn (L.W.); junfensun@dhu.edu.cn (J.S.)
[2] College of Textiles, Donghua University, Shanghai 201620, China
* Correspondence: happyjack@dhu.edu.cn (L.C.); sunbz@dhu.edu.cn (B.S.)

Received: 17 August 2020; Accepted: 22 September 2020; Published: date

Synthesis of Polystyrene

Polystyrene particles with radius of 2–3 mm was synthesized by suspension polymerization with polyvinyl alcohol (PVA) as dispersing agent, benzoyle peroxide (BPO) as the initiator, and $Na_2S_2O_3$ as termination agent. The reaction recipe is shown is **Error! Reference source not found.**. The reaction was conducted at 75 °C, 80 °C, 85 °C, 90 °C and 95 for 1 h, 1.5 h, 2.5 h. 2 h and 1.5 h, respectively.

Table S1. The reaction recipe of suspension polymerization of PS.

PVA (g/100 g styrene monomer)	BPO (g/100 g monomer)	Water (g/100 g monomer)	$Na_2S_2O_3$ (g/100 g monomer)
6	0.7	250	0.2

Rheological Properties

Table S2. Fitted parameters of the three-parameter Bird-Carreau model for PP/PS blends.

Material	η_0 (Pa·s)	n	λ (s)
PP	502	0.89	0.16
PS1	366	0.51	0.61
PS2	545	0.83	0.11
PS3	1578	0.88	0.44

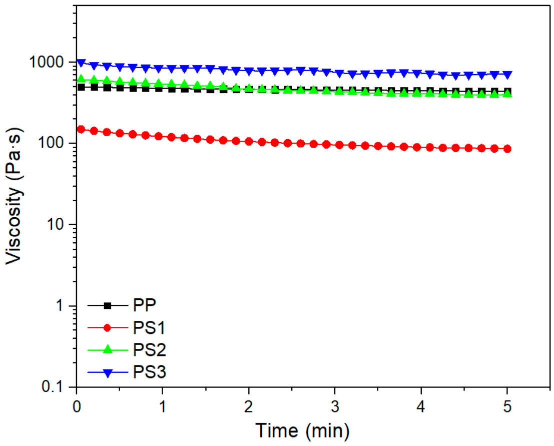

Figure S1. The shear viscosities of polymers as a function of time at 250 °C.

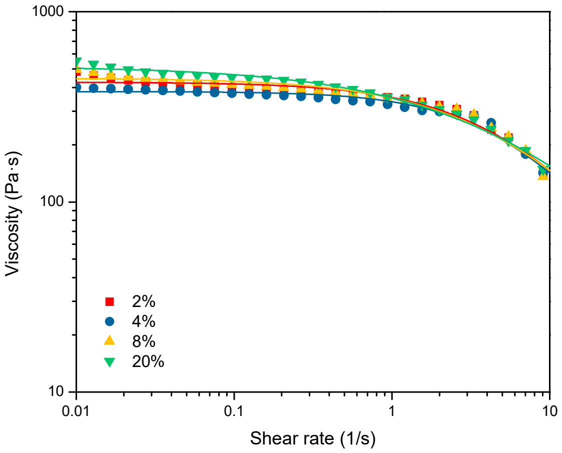

Figure S2. The shear viscosities of polymer blends as a function of shear rate at 250 °C.

© 2020 by the authors. Licensee MDPI, Basel, Switzerland. This article is an open access article distributed under the terms and conditions of the Creative Commons Attribution (CC BY) license (http://creativecommons.org/licenses/by/4.0/).
